# EnsCat: clustering of categorical data via ensembling

**DOI:** 10.1186/s12859-016-1245-9

**Published:** 2016-09-15

**Authors:** Bertrand S. Clarke, Saeid Amiri, Jennifer L. Clarke

**Affiliations:** 1 Department of Statistics, University of Nebraska-Lincoln, Lincoln, NE, USA; 2 Department of Natural and Applied Sciences, University of Wisconsin Madison, Iowa City, IA, USA; 3 Department of Food Science and Technology, University of Nebraska-Lincoln, Lincoln, NE, USA

**Keywords:** Categorical data, Clustering, Ensembling methods, High dimensional data

## Abstract

**Background:**

Clustering is a widely used collection of unsupervised learning techniques for identifying natural classes within a data set. It is often used in bioinformatics to infer population substructure. Genomic data are often categorical and high dimensional, e.g., long sequences of nucleotides. This makes inference challenging: The distance metric is often not well-defined on categorical data; running time for computations using high dimensional data can be considerable; and the Curse of Dimensionality often impedes the interpretation of the results. Up to the present, however, the literature and software addressing clustering for categorical data has not yet led to a standard approach.

**Results:**

We present software for an ensemble method that performs well in comparison with other methods regardless of the dimensionality of the data. In an ensemble method a variety of instantiations of a statistical object are found and then combined into a consensus value. It has been known for decades that ensembling generally outperforms the components that comprise it in many settings. Here, we apply this ensembling principle to clustering.

We begin by generating many hierarchical clusterings with different clustering sizes. When the dimension of the data is high, we also randomly select subspaces also of variable size, to generate clusterings. Then, we combine these clusterings into a single membership matrix and use this to obtain a new, ensembled dissimilarity matrix using Hamming distance.

**Conclusions:**

Ensemble clustering, as implemented in R and called EnsCat, gives more clearly separated clusters than other clustering techniques for categorical data. The latest version with manual and examples is available at https://github.com/jlp2duke/EnsCat.

## Background

The idea of clustering is to group unlabeled data into subsets so that both the within-group homogeneity and the between-group heterogeneity are high. The hope is that the groups will reflect the underlying structure of the data generator. Although clustering continuous data can be done in a wide variety of conceptually distinct ways there are generally far fewer techniques for categorical data. Probably the most familiar methods are *K*-modes [1], model-based clustering (MBC) [2], and various forms of hierarchical clustering. *K*-modes is *K*-means adapted to categorical data by replacing cluster means with cluster modes. MBC postulates a collection of models and assumes the clusters are representative of a mixture of those models. The weights on the models and the parameters in the model are typically estimated from the data. Hierarchical clustering is based on choosing a sense of distance between points and then merging data points or partitioning the data set, agglomerative or divisive, respectively. The merge or partition rules in a hierarchical method must also be chosen. So, hierarchical clustering is actually a large collection of techniques. Other more recent approaches include ROCK [3], which is based on a notion of graph-theoretic connectivity between points, and CLICK [4], which is based on finding fully connected subgraphs in low dimensional subspaces.

Ensembling is a general approach to finding a consensus value of some quantity that typically gives better performance than any one of the components used to form it. In the clustering context, this means that we should be able to take a collection of clusterings and somehow merge them so they will yield a consensus clustering that is better than any of the individual clusterings, possibly by separating clusters more cleanly or having some other desirable property. Thus, in principle, ensembling can be used on any collection of clusterings, however obtained.

There are many techniques by which the ensembling of clusters can be done and many techniques by which clusters to be ensembled can be generated. For instance, in the case of continuous data, four alternative methods for ensembling clusters are studied in [5]. They can also in some cases be applied to categorical data. The best of the four seems to be a form of model based clustering that rests on discretizing a data set, imputing classes to the various clusters from a collection of clusterings, and modeling the imputed classes by a mixture of discrete distributions. These methods were exemplified on five low dimensional data sets (maximum dimension 14) and even though the model based approach was overall best, the other methods were often a close second and in some cases better.

More recently, [6] developed a consensus clustering based on resampling. Any clustering technique can be repeatedly applied. Essentially, the proportion of times data points are put in the same cluster defines a collection of pairwise consensus values which are used to form *K* consensus clusters for given *K*. The ensembling method used in [6] does not seem to have been compared to the methods studied in [5]. In addition, most recently, the R-package CLUE (most recent instantiation 2016) ensembles clusters using a different and more general concept of distance than in [5] or [6] although neither of those may be regarded as special cases of CLUE.

By contrast, to ensemble clusterings, we ensemble dissimilarity matrices rather than cluster indices and we assume the data are categorical. Thus, our method is again different from those that have been presented. Our method starts by forming an incidence matrix *I* summarizing all of them. The number of columns in *I* is the number of clusterings, say *B*; each column has *n* entries, one for each data point. The (*i,j*) entry of *I* is the index of the cluster in the *j*-th clustering to which *x*_*i*_ belongs. The Hamming distances between pairs of rows in the incidence matrix effectively sums over clusterings – combining their effects – and gives a distance between each pair of data points. These distances are the entries in the ensembled dissimilarity matrix that we use in our approach. This form of ensembling is described in detail in [7]. Note that the individual clusterings that go in to forming the ensembled dissimilarity matrix are unrestricted; they may even be of different sizes.

We have used Hamming distance with equal weights since it is natural for discrete data when the principle of insufficient reason applies^1^ – it corresponds to classification loss which assumes the values are categorical rather than discrete ordinal and does not weight any one categorical variable more or less than any other. Implicitly Hamming distance also assumes that all distances between values of the same random variable are the same. Hence it is a good default when no extra information is available. Indeed, it is well known that different senses of distance correspond to different clustering criteria. Determining which sense of distance is appropriate for a given setting is a difficult and general problem. It is beyond the scope of this paper which is merely to present the software.

Given this, the question is which base clusterings to ensemble. Recall that with categorical data the measurements on a subject form a vector of discrete unordered values. Here, we will assume there is a uniform bound on the number of values each variable can assume. Data satisfying this condition is common in genetics because the nucleotide at a location is one of four values. We have ensembled a variety of clusterings generated by various methods. For instance, we have ensembled *K*-modes clusterings, model based clusterings (also called latent class clustering in some settings), and hierarchical clusterings using Hamming distance and several different linkages.^2^ As seen in [7], the best clustering results seem to emerge by i) generating hierarchical clusterings using Hamming distance and average linkage, ii) combining them into the ensembled dissimilarity matrix (again using Hamming distance), and iii) using hierarchical clustering with, say, average linkage as defined by the ensembled dissimilarity matrix. Variants on this procedure also seem to work well, e.g., complete linkage gives similar performance to average linkage and ensembling reduces the chaining problem (see [8]) associated with single linkage. Metrics other than Hamming distance may give better or worse results, but we have not investigated this because Hamming distance is such an intuitively reasonable way to assess distances between categorical vectors.

This procedure outperforms *K*-modes because, when the data are categorical, the mean is not well-defined. So, using the mode of a cluster as its ‘center’ often does not represent the location of a cluster well. Moreover, *K*-modes can depend strongly on the initial values. Using summary statistics that are continuous does not resolve this problem either; see [9] for an example.

Our procedure outperforms MBC because MBC relies on having a model that is both accurate and parsimonious – a difficult input to identify for complex data. Indeed, if we know so much about the data that we can identify good models, it must be asked why we need clustering at all – except possibly to estimate parameters such as model weights. As a separate issue MBC is computationally much more demanding than our method. We comment that in some cases, the ensemble clustering can be worse than simply using a fixed clustering method. This is often the case for *K*-modes and MBC. While somewhat counterintuitive, this is a well recognized property of other ensemble methods, such as bagging, because ensemble methods typically only give improvement on average.

Overall, in [7] our method was compared to 13 other methods (including model based clustering) over 11 real categorical data sets and numerous systematic simulation studies of categorical data in low and high dimensional settings. The theory established suggests that ensemble clustering is more accurate than non-ensembled clusterings because ensembling reduces the variability of clustering. Our finding that the method implemented here is ‘best’ is only in an average sense for the range of problems we examined among the range of techniques we tested. In all these cases, our ensembled method was the best, or nearly so, and its closest competitors on average were non-emsembled hierarchical methods that also used Hamming distance as a dissimilarity. Thus, in the present paper, we only compare our ensemble method with its non-ensembled counterpart.

At root, our method generates an ensembled dissimilarity matrix that seems to represent the distances between points better than the dissimilarity matrices used to form it. The result, typically, is that we get dendrograms that separate clusters more clearly than other methods. Thus, simply looking at the dendrogram is a good way to choose the appropriate number of clusters.

To fix notation, we assume *n* independent and identical (IID) outcomes **x**_*i*_, *i*=1,…,*n*, of a random variable *X*. The **x**_*i*_’s are assumed *J*-dimensional and written as (*x*_*i*1_,…,*x*_*iJ*_) where each *x*_*ij*_ is categorical and assumes no more than, say, *M* values. We consider three cases for the value *J*: Low dimension, i.e., *n*≫*J*, high dimension, i.e., *J*≫*n*, and high dimension but unequal, i.e., different *x*_*i*_’s can have different *J*’s and all the *J*’s are much larger than *n*. We implemented our procedure for these three cases in an R package, entitled EnsCat. As will be seen, the basic technique is for low dimensional data but scales up to high dimensional data by using random selection of subspaces and increasing the size of the ensemble. This is extended to data vectors that do not have a common length by using multiple alignment. We present these cases below.

## Implementation

We implemented our methods in the R statistical language that is free and open source [10]. So, our package does not require any other software directly. Since R is already widely used by researchers, we limit our presentation below to the functions we have defined in Enscat. The one exception to this is that if one wants to use our software on unequal length data e.g., genome sequences, the data points must be aligned and our software is compatible with any aligner. Several aligners are available in R, however, they have long running times for genome length data. As noted below, we convert categorical data values such as {*A,T,C,G*} to numerical values such as 1,2,3,4 because R runs faster on numerical values than on character values, and numerical values require less storage capacity.

When *J* is large, our methodology uses a random subspace approach to reduce the dimension of the vectors being clustered. We do this by bootstrapping. Given the set {1,…,*J*} we choose a sample of size *J* with replacement, i.e., we take *J* IID draws from {1,…,*J*}. Then we eliminate multiplicity to get a set of size *J*^∗^≤*J* of distinct elements. This procedure can be repeated on the set with *J*^∗^ elements, if desired, to get a smaller set of distinct dimensions. Since the dimensions of the subspaces are random, they will, in general, be different. This allows our procedure to encapsulate a large number of relationships among the entires in the *x*_*i*_’s. As a generality, ensemble methods are robust by construction because they represent a consensus of the components being ensembled. This is formally true for random forests in classification contexts, [11], and partially explains why ensemble methods are not always optimal. Sometimes a single component routinely outperforms ensembles of them; this seems to be the case with *K*-modes and MBC.

## Results and discussion

### Low dimensional categorical data

The package Enscat includes functions for implementing *K*-modes, hierarchical clustering methods, and our ensemble clustering method. It can also call routines for MBC. To show how this works, here we use the data set USFlag as an example. This dataset contains information about maritime vessels in the U.S.-Flag Privately-Owned Fleet and can be downloaded from the United States Department of Transportation site for United States Maritime Administration data and statistics [12]. USFlag has sample size *n*=170 and each observation has 10 categorical variables containing information about vessel operator, vessel size and weight, and vessel type (Containership, Dry Bulk, General Cargo, Ro-Ro, and Tanker, denoted as 1 through 5). The data are stored in USFlag$obs and the vessel types are stored in USFlag$lab. Once the Enscat package has been downloaded and installed, *K*-modes clustering can be done in R by using the commands

1 $$\begin{array}{@{}rcl@{}} &&\texttt{library(EnsCat)}\\[-3pt] &&\texttt{data(USFlag)} \\[-3pt] &&\texttt{kmodes(USFlag\$obs, k=5,k2=1:5)}  \end{array} $$

The second argument in the function kmodes,k=5, is the number of clusters *K*-modes should output. The third argument is the specification of the initial modes. Here, 1:5 means the first five data points should be taken as the initial modes. As recognized in [1], *K*-modes is sensitive to initial values, possibly leading to instability and inaccuracy.

Hierarchical clustering has attracted more attention than *K*-modes since it provides a nested sequence of clusterings that can be represented as a tree or dendrogram. This technique requires a matrix specifying the distances between data points; such a matrix can be calculated using Hamming distance. The following commands generate a dendrogram using Hamming distance and average linkage.



**Fig. 1:**
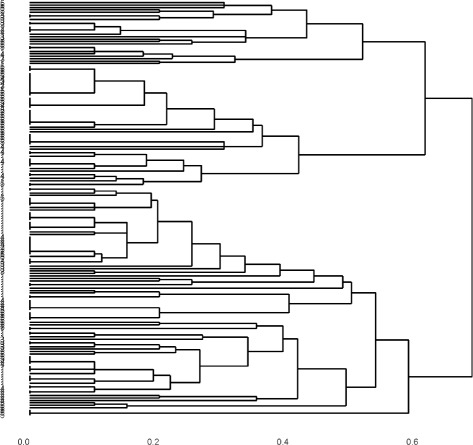
Dendrogram generated by average hamming distance on USFlag data

The first command generates the *n*×*n* matrix in which the (*i,j*)-th entry is (1/*J*)*H*(*x*_*i*_,*x*_*j*_)∈[0,1] where *H* is the Hamming distance between its arguments. The second command tells R to regard distham0 as a matrix of distance. Taken together, the third and fourth commands produce and plot the dendrogram for hierarchical clustering using distham0 and average linkage. The command ggdplot is a convenience wrapper for the function ggdendrogram in the package ggdendro which automates the plotting of a rotated dendrogram with user specified leaf labels and plot title. The results are shown in Fig. 1.

By contrast, our ensembling algorithm is the following.

Ensemble clustering of USFlag can be done by the following.



**Fig. 2:**
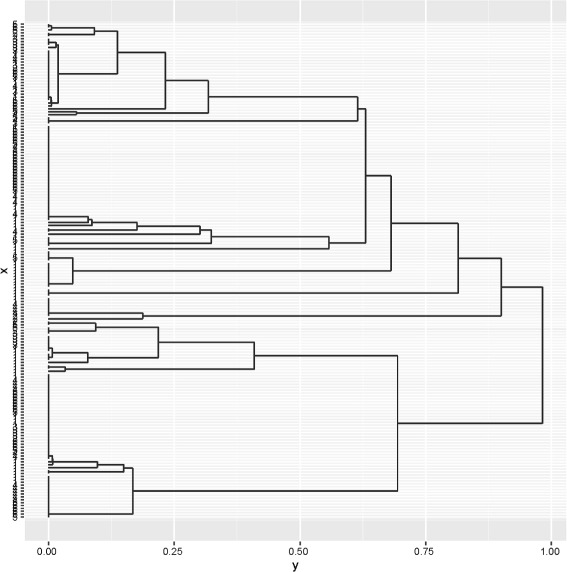
Dendrogram generated by the ensemble method on USFlag data



The first command uses Benhc, one of two functions in Enscat that implement our new method. This generates the ensembled dissimilarity matrix *T* by combining *B*=200 hierarchical clusterings using Hamming distance and average linkage, each generated by Steps 1, 2, and 3 in Algorithm 1. (As a generality, average linkage was found to perform well in this context, see [7].) The second command runs a hierarchical clustering using *T* and average linkage. hclust is a function in R from the package stats that can be used to make a dendrogram. The third command generates a plot of the ensembled dendrogram, with a grayscale grid in the background to help gauge the length of each lifetime; see Fig. 2. In contrast with Fig. 1, the ensembling gives longer ‘lifetimes’, i.e., the vertical lines connecting to the individual data points. Longer lifetimes mean that the clusters are separated more clearly. We found this to be the typical effect of ensembling.

Estimating the correct number of clusters, *K*_*T*_, is a difficult problem. Several consistent methods are known for continuous data, see [13] for a discussion and comparison of such techniques. In the categorical data context, some techniques such as *K*-modes and MBC require *K* as an input and in practice the user chooses the value of *K* that gives the most satisfactory results. For hierarchical clustering, *K* need not be pre-assigned; it can be inferred, at least heuristically, from the dendrogram. When ensembling separates the clusters more clearly inferring *K* may be easier. In particular, it can be seen from the increased number of long lifetimes in the dendrogram of Fig. 2 relative to the number of long lifetimes in the dendrogram of Fig. 1 that the ensembling visibly improves the separability of the clusters leading to fewer, more distinct clusters. Thus, simply looking at the dendrogram may be a good way to choose the appropriate number of clusters.

**Fig. 3:**
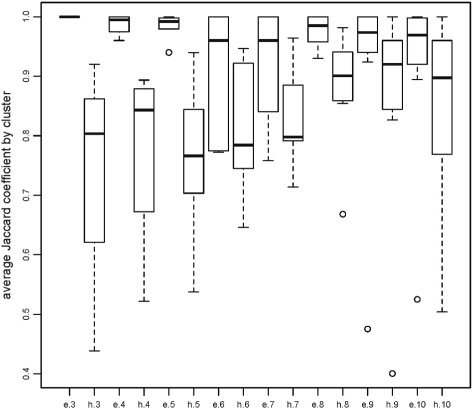
Jaccard resampling stability analysis for the USFlag data. The labels indicate the type of clustering and the number of clusters, e.g., e.3 is ensembled hierarchical clustering with 3 clusters while h.3 is non-ensembled hierarchical clustering with 3 clusters

This observation is heuristic but is supported by formal stability computations under perturbation indices, for instance. One established approach is due to [14]. The idea is to generate a range of clusterings of various sizes. Then, for each clustering, reclustering *B* bootstrap samples from it evaluating the Jaccard coefficient by cluster for each clustering. Higher values of the Jaccard coefficients for the clusters indicate higher reproducibility under bootstrapping and hence high stability. We have applied this procedure using the function clusterboot in the R-package fpc. The result is in Fig. 3. For *K*=3,…,10, the boxplots of the Jaccard coefficient between the original clusters and the clusterings based on the resampled data are plotted. The notation ‘e’ on the horizontal axis indicates the ensembled version; ‘h’ indicates the (not ensembled) hierarchical version. For each *K* from 3 to 10, the ensembled version is strikingly more stable. Although not shown here, the same qualitative behavior can be observed if the adjusted Rand index is used. On the other hand, the behavior is similar but not identical if the unadjusted rand index is used. So, there may be some dependence on the exact form of data point perturbation method.

### High dimensional categorical data: fixed length

Our method extends to high dimensional fixed length data by clustering on random subspaces, i.e., random selections of the categorical variables of comparatively smaller dimension, using Hamming distance and average linkage. Taken together these clusterings give an $\mathcal {I}$ as in Algorithm 1 and Steps 5 and 6 can be performed. As can be seen by comparing Algorithms 1 and 2, the only methodological difference between our treatment of low and high dimensional categorical data is the clustering on random subspaces.



It is seen that the $X_{b}^{*}$’s contain independently chosen subsamples of possibly different sizes of the *J* variables so that all variables have the same chance of inclusion. The commands for implementing Algorithm 2 are a special case of those given in the next subsection (i.e., in Step 1 run enhcHi with type=1)^3^. If *J* is so large that the output of Step 2 results in an unacceptably long running time for Step 4, a second, or even third, level of boostrapping can be used (i.e., interactively bootstrap the bootstrap sample to further reduce the number of variables). Enscat does implement a double bootstrap in the high dimensional case; see the example code for Algorithm 2.

### High dimensional categorical data: non-fixed length

Our method extends to non-fixed length high dimensional data by ‘filling in’ missing variables by alignment and then using random subspace clustering as described in the last subsection. As an example we generate an ensemble clustering of complete viral genomes from the Family Rhabdoviridae. According to the Virus Pathogen Database and Analysis Resource (ViPR)([15]), Rhabdoviridae is divided into 12 genera and for ease of exposition we have limited our analysis to the data set containing all distinct and complete genomes from those genera with less than 40 complete genomes (9 of 12 genera).

The 9 relevant genera of Rhabdoviridae are, namely, Cytorhabdovirus, Ephemerovirus, Novirhabdovirus, Nucleorhabdovirus, Perhabdovirus, Sigmavirus, Sprivivirus, Tibrovirus, and Tupavirus, with 5, 10, 16, 10, 1, 3, 5, 1, and 2 genomes, respectively. The viruses belonging to these genera came from different hosts, namely, Alfalfa, Cattle, Drosophila, Eel, Fish, Garlic, Midge, Mosquito, Eggplant, Taro, Trout, and Unknown. In the dendrograms each sample is identified by the first two letters of the genus and the first three letters of the host (e.g., Cytorhabdovirus from Alfalfa is labeled Cy.Alf). The genomes have lengths between 10,845 and 16,133 base pairs. In principle, we could have included incomplete genomes and filled in the missing data by imputation via alignment. For simplicity we did not do this.

To cluster categorical vectors of different lengths, the first step is to preprocess the data using a multiple alignment approach so all the vectors have the same length. This is done by including an extra value, say *ϕ*, that is inserted in various locations so that sequences of nucleotides match as closely as reasonably possible. There are several programs that do multiple alignment and they can give different equalized lengths depending on the exact rules followed for inserting *ϕ*. We used MAFFT-7 [16] but any aligner would be compatible with our software, although different aligners might give different results. We stored the aligned data in a file called ‘rhabdodata’ and pulled it into R using data(“rhabdodata”). Now, the data take values in {*A,T,C,G*,*ϕ*}. For efficiency, this categorical data is converted to numerical data. The Enscat command CTN does this conversion; {*A,T,C,G*,*ϕ*} in rhabdodata are replaced with {1,2,3,4,*NA*}. R recognizes NA as missing data so this does not increase Enscat’s running time.

Hierarchical clustering on rhabdodata using Hamming distance and average linkage is given by



**Fig. 4:**
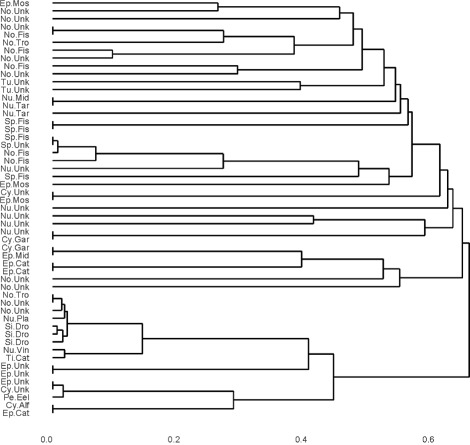
Dendrogram generated by average hamming distance on rhabdodata via *DU*(2,8)

This is the same code as for low dimensional categorical data. The resulting dendrogram is in Fig. 4.

The dendrogram of the ensemble clustering for rhabdodata using hierarchical clustering with average linkage and the matrix *T* generated by the *B* clusterings of the random subspaces can be obtained using the following commands.



**Fig. 5:**
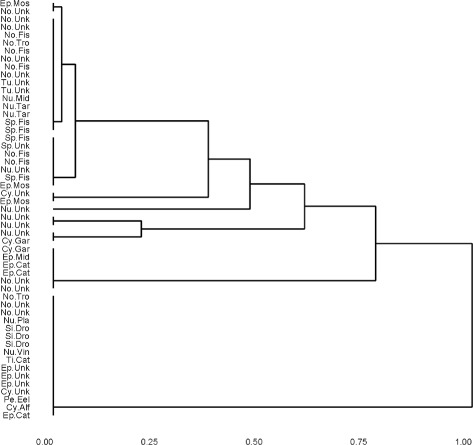
Dendrogram generated by ensembling clusterings of random sizes on rhabdodata via *DU*(2,8)

enhcHi is the Enscat function that generates $\mathcal {I}$ for the equalized length vectors; it stands for ensembling hierarchical clusterings of high dimension. In this example, *B*=100 random subspaces are chosen and the values *K*_*b*_ are chosen according to a DUnif [2, 8]. We chose 8 because $8 \approx \sqrt {53}$ and *n*=5+10+16+10+1+3+5+1+2=53. The argument type=2 specifies a double bootstrap procedure for variable selection. The result is in Fig. 5.

**Fig. 6:**
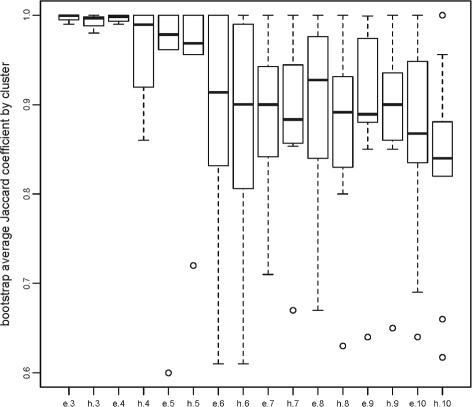
Jaccard resampling stability analysis for the rhabdoviridae data. The labels indicate the type of clustering and the number of clusters, e.g., e.3 is ensembled hierarchical clustering with 3 clusters while h.3 is non-ensembles hierarchical clustering with 3 clusters

We note that Fig. 5 improves on Fig. 4 in the same sense as Fig. 2 improves on Fig. 1. That is, the dendrograms from the ensemble methods have an increased number of long lifetimes relative to short lifetimes suggesting increased stability. Again, this is reflected in the Jaccard coefficients for the various choices of *K*, see Fig. 6. For *K*=3,…,8 it is clear that the ensemble method gives higher stability than the hierarchical method though the improvement is not as much as in the low dimensional case for reasons discussed earlier. As a point of interest, note that stability indices can themselves become unstable when there is too little data per cluster. This is seen for *K*=9,10 in Fig. 6.

**Fig. 7:**
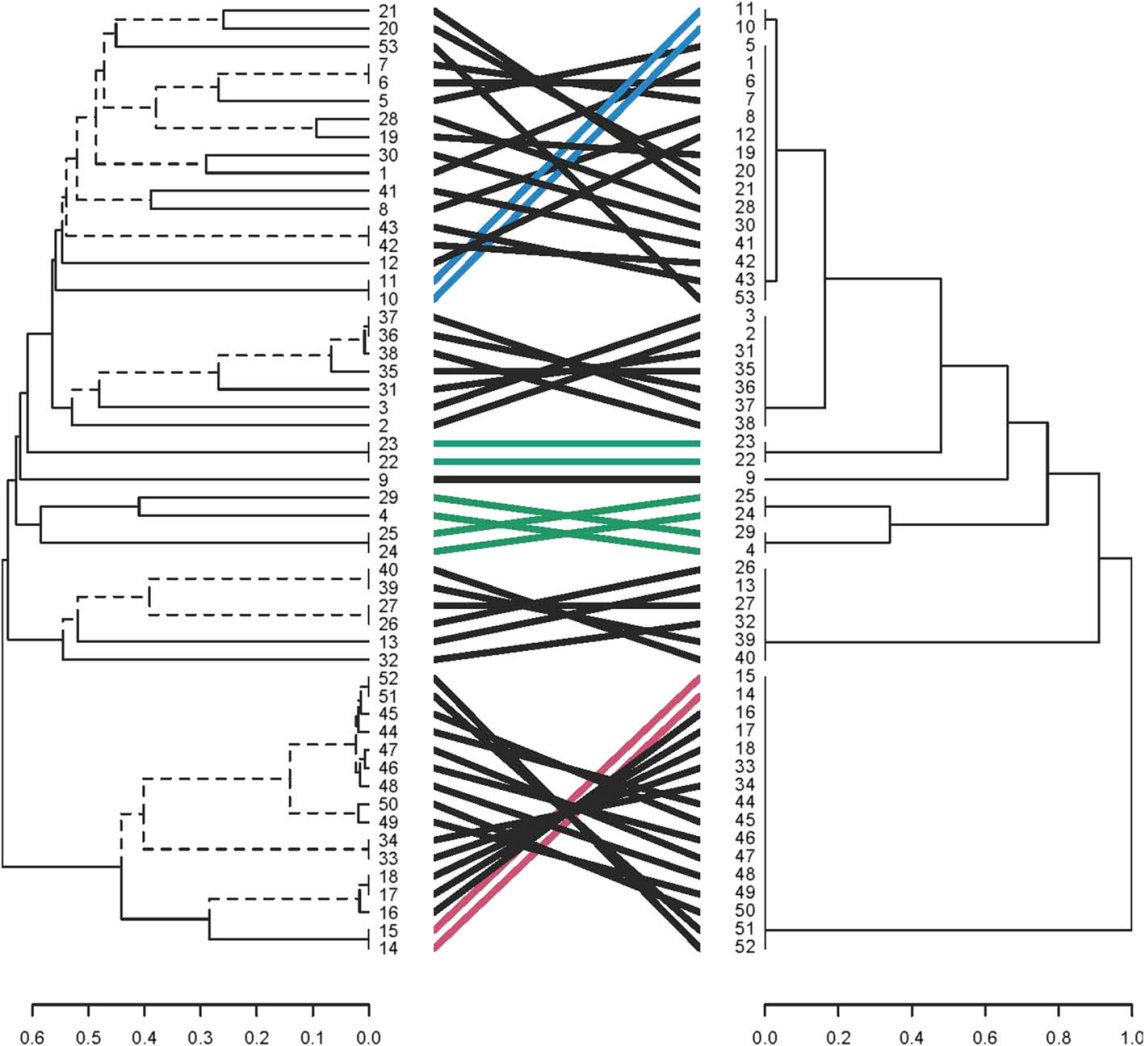
A tanglegram of the clusterings shown in Figs. 4 and 5. This visualizes the amplification and clarification of the cluster structure provided by our ensembling approach. The same samples in both clusterings are connected by lines; branches that appear only in one clustering are denoted by dashed lines

An alternative way to visualize the improvement provided by ensembling is shown in the ‘tanglegram’ in Fig. 7. The left hand dendrogram shows the average linkage hierarchical clustering under Hamming distance, while the right hand dendrogram shows the ensembled version of clusterings of this form. This figure was generated by a simple command,

tangle(hc0, hc1)

Colored lines connect the same data points in the two different clusterings; the dashed lines identify branches that exist in only one of the two dendrograms. It is visually apparent that the ensembling collapses many branches into single clusters. That is, the ensembling amplifies and simplifies the underlying cluster structure, so that the clusters are more readily discernible. For a nice exposition of tanglegrams and other visualizations of dendrograms see Galili (2015) and the associated package dendextend [17].

**Fig. 8:**
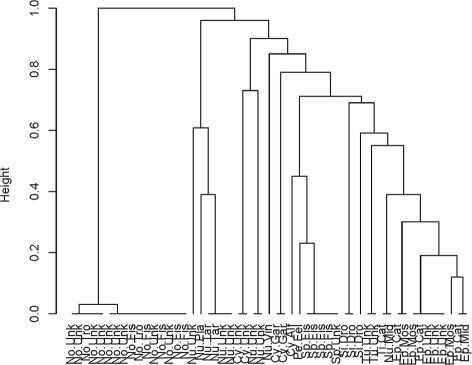
Dendrogram generated by ensembling clusterings of random sizes on rhabdodata via *DU*(2,20)

To see the effect of the range of the number of clusters, Fig. 8 shows the result of using our ensemble method drawing the *k*_*b*_’s from DUnif(2,20). It is seen that by increasing the range of the *K*_*b*_’s, the sensitivity of the ensemble clustering to the data increases. This is no surprise because the range of clusterings has increased thereby decreasing the stability and this effect is larger in high dimensions than small. While we can generate a Jaccard stability assessment for the dendrograms in Figs. 5 and 8, we have seen that the Jaccard coefficient matches our visual intuition well so it is enough to argue that the degree of sensitivity in Fig. 8 is a little too high. This follows from noting that the number of long lifetimes in the dendrogram has decreased visibly. In practice, a user should test several ranges for the *K*_*b*_’s and choose the results that produce the clearest separation between clusters. Although informal, this is a common approach to selecting a clustering and works typically as well as many formal methods.

## Simulation

For the sake of completeness, we give a simulated example of our method for equilength categorical vectors, both low and high dimensional. We generated data sets from different parameterizations and structures of multinomial distributions,100 data sets at each setting. The parameter values in the data generating model were fixed according to a simulation scheme (full factorial design) that allowed for examining the impact of several aspects of the clustering procedure:

- number of observed variables, taken as 12 and 100
- sample sizes, taken as 100, 300, 1000
- number of categories, taken as 2, 3, 4, 8
- number of clusters, taken as 3, 5, 7
- size of clusters, taken as all equipopulated or representing a range of differences of proportions of the data
- expected cluster separations, ranging from all categories equi-likely to some categories likely and some not very likely

We generated 24 data sets at each of the three sample sizes, thus 72 data sets in total. Of the 24 data sets at a given sample size, 12 had 12 variables and 12 had 100 variables. In the case of 12 variables, three were binary, three were ternary, four were quaternary, and two were octonary. In the case of 100 variables, all were quaternary. For each of the 72 data sets, we tested three, five, and seven clusters taken as true. Hence we generated 432 clusterings, half using the hierarchical method based on Hamming distance and half using our ensemble method. We then used the Jaccard stability method as before to compare the 216 clusterings under one method to their respective 216 clusterings under the other method.

While many implications can be drawn from this simulation we focus on the fact that in all cases the difference between the median Jaccard index for the ensemble method was strictly greater than the median Jaccard index for the hierarchical method. Indeed, for sample size 100, the average median increase in stability was.174. The corresponding increases for sample sizes 300 and 1000 were.296 and.399, respectively. That is, the stability of our method over hierarchical clustering increased substantially with sample size. We comment that in the absence of knowledge of the skewness of the distribution of the Jaccard coefficient we have defaulted to the median, a slightly less efficient measure of location than the mean but qualitatively giving the same results for the sample sizes we have used.

As a separate point, the median improvement of ensemble clustering over hierarchical clustering in terms of Jaccard stability decreases with dimension. For the case of 12 dimensions, the increase in stability is.327 and for 100 dimensions it is.252. This is no surprise since it is well known that as dimension increases, clustering becomes increasingly unstable to the point where, in the limit of large dimensions, all clusterings are equally valid. This counter-intuitive result was first shown in [18] and a characterization established in [19]. Thus, in the limit of high dimensions, increases in stability are harder to achieve so even small ones may be important.

## Conclusion

Here we have described software that can cluster high dimensional categorical data points that have unequal lengths. The code is implemented in R, a well-known programming language. Our method is based on ensembling, random subsets, and pre-processing data (if necessary) by using an aligner.

There are two pragmatic benefits of the methodology built into our software. First, it is clearly seen that ensembling clusterings, at least when done well, gives results that are no worse and usually much better that using an individual clustering. The main way this is accomplished is by the reduction of ‘variability’. This is seen in the longer lifetimes, the increased stability, and the elimination of many of the dashed lines in the tanglegram. This parallels the well-established principle that ensembling generally gives better results when applied to classification and regression problems – also by reducing variability. A particularly clear instance of this is seen in the theory behind bagging, see [20].

Second, the main contribution of the methodology and software is to put the emphasis where it is more useful, namely on the construction of good candidate clusterings for categorical data. As can be seen, evaluating clustering stability is a well developed field with many methods that are typically accepted as intuitively reasonable. We have used the Jaccard index but could have equally well used other resampling based indices such as the adjusted Rand, the variation in information, etc. There are other classes of stability methods, e.g., Bayesian, but these are beyond our present scope. Each specific technique has its strengths and weaknesses that become apparent in extreme cases (very small clusters, clusters that are close together, etc.) but outside of these cases the various methods for stability do not widely differ.

It is worth commenting that dimension reduction methods are sometimes considered a competitor to ensemble methods. Indeed, feature selection is one of the main ways practitioners try to evade the Curse of Dimensionality. The idea is to reduce the dimension of the vectors to be clustered by identifying relatively few functions of the variables thought to characterize the clusters. Evidence has accumulated that feature selection does not work very well on continuous data – absent extensive knowledge about which features are relevant – see [21, 22], and [23]. Methods such as [24] that try to weight explanatory variables adaptively are also known not to perform well. Moreover, if generic methods for obtaining features, e.g., PCA, are used with categorical data, the computing demands become infeasible. Since techniques based on feature selection are even harder to devise and compute for discrete data, feature selection does not seem a promising approach to high dimensional clustering of categorical data. Otherwise put, generic techniques such as ours that do not rely on extensive subject-matter knowledge are often the only available techniques.

Generating good categorical clusterings to assess should be the general focus of methodological development and exploration. This is hampered by the fact that (i) clustering, like any model selection type of methodology, tends to require large amounts of data to be effective and (ii) by the fact that high dimensional data is qualitatively different from low dimensional data. This is so because the concept of distance has an increasing impact on the spatial relationships of the data points making stability more difficult to achieve. Hence, smaller increases in stability are overall more important in high dimensions than low. Indeed, it has been argued that finite dimensional intuition becomes ever more inappropriate as dimension increases; see [25] who argues that in the limit of high dimensions ultrametric distances between points are more representative than Euclidean distances. Nevertheless, our method seems to be flexible, capable of generating plausible clusterings when used reasonably, and amenable to stability assessments for finite dimensions.

We conclude by noting that even in the simplest case – clustering low dimensional categorical data having equal lengths – no previous method can be regarded as well-established. However, in [7], we have argued theoretically and by examples that the method implemented by our software performs better on average than many other clustering methods in settings where other methods exist. We have also argued that in the case of fixed length high dimensional clustering our method outperforms mixed weighted *K*-modes, a technique from [26]. In the case of non-fixed length high dimensional data, we have compared our method to phylogenetic trees developed from biomarkers. Our method appears to give results that are equally or slightly more accurate and more generally attainable since they do not rest on biological information that is often not available.

## Availability and requirements

**Project name:** EnsCat. **Project home page:**https://github.com/jlp2duke/EnsCat**Operating systems:** Windows, OS X. **Programming language:** R ≥ 3.2.4. **Other requirements:** aligner (for unequal length data). **License:** GNU, GPL.**Any restrictions to use by non-academics:** None.

## Endnotes

^1^ The principle of insufficient reason states that one should assign a uniform value across elements in the absence of reason to do otherwise.

^2^ In hierarchical clustering a ‘linkage’ function must be defined. A linkage function represents a distance or sum of distances from any given point set to another point set. Single linkage means the shortest distance between the two point sets. Complete linkage means the longest distance between two point sets. Average linkage means the average of all the distances between the points in the two sets. There are other linkage functions that are used but these two are the most common.

^3^ The commands are given in the manual at https://github.com/jlp2duke/EnsCat.

## Abbreviations

DUnif: Uniform distribution
IID: Independent and identically distributed
MBC: Model based clustering
PCA: Principal components analysis
ViPR: Virus Pathogen Database and Analysis Resource
